# A spiral microfluidic device for rapid sorting, trapping, and long-term live imaging of *Caenorhabditis elegans* embryos

**DOI:** 10.1038/s41378-023-00485-4

**Published:** 2023-02-21

**Authors:** Peng Pan, Zhen Qin, William Sun, Yuxiao Zhou, Shaojia Wang, Pengfei Song, Yong Wang, Changhai Ru, Xin Wang, John Calarco, Xinyu Liu

**Affiliations:** 1 Department of Mechanical and Industrial Engineering, University of Toronto, 5 King’s College Road, Toronto, Ontario M5S 3G8 Canada; 2 Upper Canada College, 200 Lonsdale Road, Toronto, Ontario M4V 1W6 Canada; 3 School of Advanced Technology, Xi’an Jiaotong-Liverpool University, 111 Ren’ai Road, Suzhou, 215000 China; 4 School of Electronic and Information Engineering, Suzhou University of Science and Technology, Suzhou, 215009 China; 5 Department of Mechanical and Aerospace Engineering, Jilin University, Changchun, 130012 China; 6 Department of Cell & Systems Biology, University of Toronto, 25 Harbord St, Toronto, Ontario M5S 3G5 Canada; 7 Institute of Biomedical Engineering, University of Toronto, 164 College Street, Toronto, Ontario M5S 3G9 Canada

**Keywords:** Engineering, Materials science

## Abstract

*Caenorhabditis elegans* embryos have been widely used to study cellular processes and developmental regulation at early stages. However, most existing microfluidic devices focus on the studies of larval or adult worms rather than embryos. To accurately study the real-time dynamics of embryonic development under different conditions, many technical barriers must be overcome; these can include single-embryo sorting and immobilization, precise control of the experimental environment, and long-term live imaging of embryos. This paper reports a spiral microfluidic device for effective sorting, trapping, and long-term live imaging of single *C. elegans* embryos under precisely controlled experimental conditions. The device successfully sorts embryos from a mixed population of *C. elegans* at different developmental stages via Dean vortices generated inside a spiral microchannel and traps the sorted embryos at single-cell resolution through hydrodynamic traps on the sidewall of the spiral channel for long-term imaging. Through the well-controlled microenvironment inside the microfluidic device, the response of the trapped *C. elegans* embryos to mechanical and chemical stimulation can be quantitatively measured. The experimental results show that a gentle hydrodynamic force would induce faster growth of embryos, and embryos developmentally arrested in the high-salinity solution could be rescued by the M9 buffer. The microfluidic device provides new avenues for easy, rapid, high-content screening of *C. elegans* embryos.

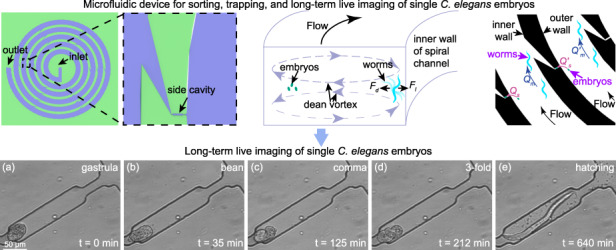

## Introduction

As a small model organism, the nematode worm *C. elegans* has many advantages, such as small size, a short life cycle, a transparent body, and a significant genomic overlap (~60–80%) with humans^1,2^. Because of these merits, *C. elegans* is widely used in biological and medical studies such as neurodegenerative disease research^3,4^, gene screening^5^, drug discovery^6^, and many others^7–11^. The developmental stages of *C. elegans* include the embryonic stage, the four larval stages (L1-L4), and the adult stage. At each stage, *C. elegans* shows distinct features^12^. For instance, the size of *C. elegans* increases during the four larval stages and reaches the maximum at the adult stage. Additionally, each stage of *C. elegans* shows varying cuticle composition^13^ and different nervous system^14^. Among these stages, the *C. elegans* embryo is an excellent model system to study biological mechanisms such as cellular processes and developmental regulation at a very early stage^15^. However, despite the advantages of the embryonic stage for biological research, such as short developmental time and immobile nature, long-term studies of individual *C. elegans* embryos during development are still technically challenging.

The current protocol for live imaging of embryonic development relies on embryos mounted on an agar plate, and the sample preparation process is tedious and skill dependent. In addition, this conventional protocol does not allow for long-term live imaging over the complete course of embryogenesis and precise control of surrounding environmental conditions. Recently, microfluidic devices fabricated from polydimethylsiloxane (PDMS) have facilitated the handling of *C. elegans* embryos owing to their advantages such as high degree of transparency, size matching, good biocompatibility, and ease of use. A microfluidic device with several microwells was proposed to immobilize *C. elegans* embryos obtained from dissected worms for long-term live imaging^16^. Later, two similar microfluidic devices were developed to immobilize embryos using passive hydrodynamics^6,17^; in these devices, the embryos were synchronized by worm dissection or bleaching procedure. Conventional embryo synchronization methods, including worm dissection or bleaching procedures, can cause damage to embryos and generate worm debris, resulting in the clogging of microfluidic devices^17,18^. Since a mixed population consisting of embryos, larval-stage worms, and adult worms is usually obtained by culturing several adult worms on a fresh agar plate (with OP50) for 3–4 days, efficient sorting of embryos is still a difficult task due to the small sizes of the embryos and the worms.

Several microfluidic devices have been developed to sort worms at different developmental stages utilizing worms’ distinct responses to an electrical field^19–23^. However, electrical fields have been demonstrated to have a negative effect on worms due to their effect on the animal’s motility^21,22^. Nonelectrical methods were also developed for sorting worms based on their size at each developmental stage^24–29^. Size-based sorting has been demonstrated by using image processing algorithms for active identification of worm sizes^24,26^, an array of geometrically optimized pillars^29^, two rings of sorting filters with different feature sizes^28^, adjustable valves through membrane deflection^29^, and trapezoidal channels integrated into microfluidic spiral chips^30^. However, most of the developed microfluidic techniques have focused on the sorting of L1-L4 and adult worms for synchronization rather than embryos. Moreover, the introduced microfluidic designs have challenges in device fabrication, reliable operation, cost, and reusability, limiting their potential use in practical applications.

To avoid the damage caused by the conventional embryo preparation protocol and realize parallel studies of embryos under different conditions, we herein report a spiral microfluidic device for facile sorting, trapping, and long-term imaging of *C. elegans* embryos. This device is capable of automatically sorting embryos from a mixed population of *C. elegans* at different stages via Dean vortices generated inside a spiral channel, as well as trapping single sorted embryos in hydrodynamic traps on the sidewall of the spiral channel for long-term imaging. Using the proposed microfluidic device, the developmental stages of embryos from the gastrula stage to the hatching stage can be readily imaged at high resolution. As the embryos are trapped and cultured in the traps, the microenvironment surrounding these trapped embryos can be precisely controlled. To highlight this capability, we examine the response of the trapped *C. elegans* embryos to mechanical and chemical stimulation and investigate the underlying developmental mechanisms. This microfluidic device provides a new tool for facile, rapid, high-content screening of *C. elegans* embryos.

## Results and discussion

### Microfluidic device design

**Fig. 1:**
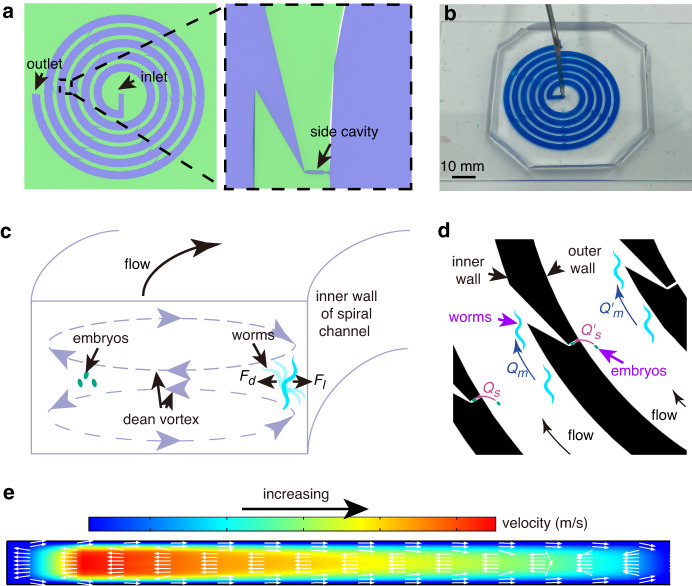
Microfluidic device for sorting and trapping worm embryos. **a** Schematic layout of the microfluidic device. **b** Photograph of the microfluidic device. **c** Schematic of inertial focusing of adult worms and embryos. **d** Schematic of trapping of focused embryos. *Q*_*s*_ represents volume flow rate along the side cavity, and *Q*_*m*_ represents volume flow rate along spiral channel. **e** Numerical analysis showing the generation of Dean vortices in a cross-section of the spiral channel

The microfluidic device consists of a PDMS microchannel layer bonded atop a glass slide. The layout of the microchannel in the PDMS layer is schematically shown in Fig. 1a, including a spiral microchannel (1.6 mm wide and 53 µm thick) and 20 side cavities (as hydrodynamic traps) uniformly distributed along the outer wall of the spiral channel (Fig. S1). Each side cavity is designed to ensure that its hydrodynamic resistance is large and that the resulting volume flow rate along the side cavity is smaller than that along the spiral channel (Fig. S2). In this case, objects close to the outer wall of the spiral channel can be captured/trapped, while objects close to the inner wall bypass the side cavity. It should be noted that the width of the front section of the side cavity is 20 µm (Fig. S1), slightly smaller than the diameter of an embryo (~30 µm), preventing captured embryos from escaping the side cavity when the flow rate is low and/or certain vibration occurs during experiments.

When a fluid carrying embryonic, larval, and adult *C. elegans* flows through the spiral channel (Fig. 1b), it experiences centrifugal acceleration along the radially outward direction, which results in the formation of two counterrotating Dean vortices in the bottom and top halves of the spiral channel cross-section^31^. Under the Dean drag and lift forces, the worms with large hydraulic diameters (L4 and adult worms) are focused close to the inner wall of the spiral channel, and embryos (with small hydraulic diameters) are focused close to the outer wall (Fig. 1c)^32^. Once the small embryos are sorted from the mixed population and focused close to the outer wall, they are trapped by the uniformly distributed side cavities through passive hydrodynamics (Fig. 1d, and Video S1). Regarding the L1 worms, even if they are brought into the side cavities, they can easily exit the side cavities via the other end under the action of hydrodynamic force, as the diameter of L1 worms (~18 μm) is close to the width of the narrowest part (15 μm; Fig. S1) of the side cavity. Numerical simulations (Fig. 1e) show that Dean vortices can still be generated in the cross-section of the spiral channel when the side cavities are distributed along the channel (Fig. S3).

### Sorting and trapping of single embryos

The ratio of the hydraulic diameter of worms/embryos to the hydraulic diameters of the channel is the major factor that determines the focused behaviors of worms/embryos in the spiral channel. To realize worms/embryo focusing, the ratio of the hydraulic diameters of the worms/embryos to that of the spiral channel should be >0.07^30,33^. *C. elegans* embryos have an oval shape with an average hydraulic radius of ~24 μm, while L1, L2, L3, L4 and adult worms have a rod-like shape with average hydraulic radii of 26 μm, 32 μm, 40 μm, 61 μm, and 78 μm, respectively. The hydraulic diameter of the channel is ~100 μm^30^. As the ratio of worms’ diameters to the hydraulic diameter of the channel is much >0.07, the focusing of *C. elegans* worms/embryos at different stages in the spiral channel can be achieved.

**Fig. 2:**
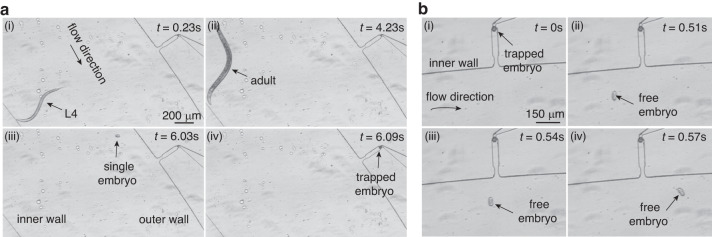
Sorting and trapping of embryos from a mixed population of worms. **a** Image sequence showing an embryo focused close to outer wall while L4 and adult worms were focused close to inner wall. **b** Image sequence showing a free embryo bypassing the occupied side cavity

A mixed population of embryos, L1 larvae, L4 larvae, and adult worms was obtained by following a standard synchronization protocol and was used for single-embryo sorting and trapping. The synchronization protocol started with a culture of 5–8 adult worms on a fresh agar plate (with OP50) for 1 day. Later, these 5-8 adult worms were removed, and the remaining embryos or larval worms were continuously maintained for another two and one-half days at 21 °C. Finally, a mixed population consisting of a large number of embryonic, L1 larvae, L4 larvae, and adult worms was obtained (Fig. S4 and Table S1). The mixed population was then loaded into the microfluidic device. The L4 and adult worms with large hydraulic diameters were successfully focused close to the inner wall of the spiral channel, while embryos were focused close to the outer wall of the spiral channel (Fig. 2a and Video S1). Once the sorted embryo moved close to an empty side cavity, it was captured by passive hydrodynamics (Fig. 2a-iii). It should be noted that as the hydrodynamic resistance of the side cavity is large, the L4/adult worms, which are focused close to the inner wall, can easily bypass the side cavity. At a flow rate of 1 mL/min in the spiral channel, 98.75% ± 2.5% (*N* = 4 devices) of side cavities successfully trapped the embryos.

**Fig. 3:**
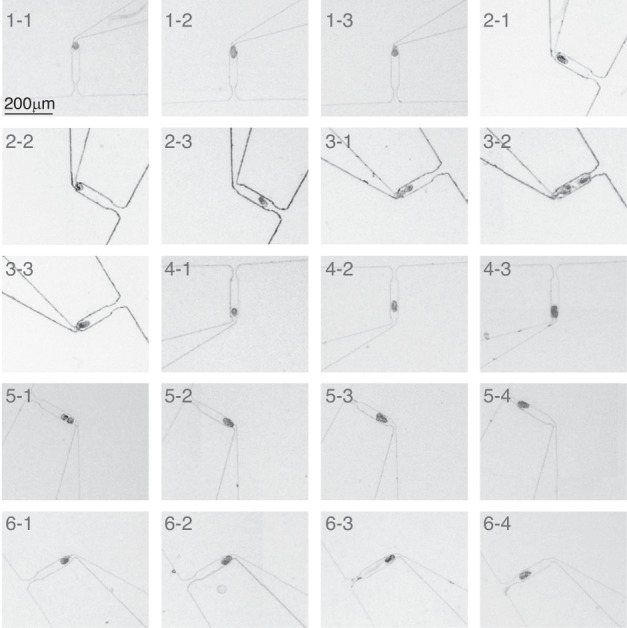
Images showing captured embryos in all side cavities of the same microfluidic device. *x-y* represents the *y*th side cavity of the *x*th array

To enable long-term live imaging of embryos, the capture of one embryo per side cavity is necessary. As shown in Fig. 2b, once an embryo occupied a cavity, other embryos bypassed the occupied cavity and were captured in subsequent empty cavities (Video S2). This allowed for the creation of an array of captured embryos for long-term imaging with registered positions. At a flow rate of 1 mL/min, 83.75 ± 2.5% (*N* = 4 devices) of the side cavities captured one and only one embryo each. Figure 3 shows the images of 20 side cavities along the spiral channel (4×, NA = 0.13) when the mixed population was loaded at a flow rate of 1 mL/min (Fig. S5). It can be seen that 17 cavities captured single embryos, while only three cavities (marked as 3–1, 3–2, and 5–1) captured two embryos. During worm loading, a low number of embryos were found to adhere to each other, which might have contributed to the capture of two embryos in the three cavities. This is because if two embryos adhere to each other, their equivalent hydraulic radius is ~29 μm, which is just slightly larger than that of a single embryo (~24 μm).

### Long-term imaging of embryos

**Fig. 4:**
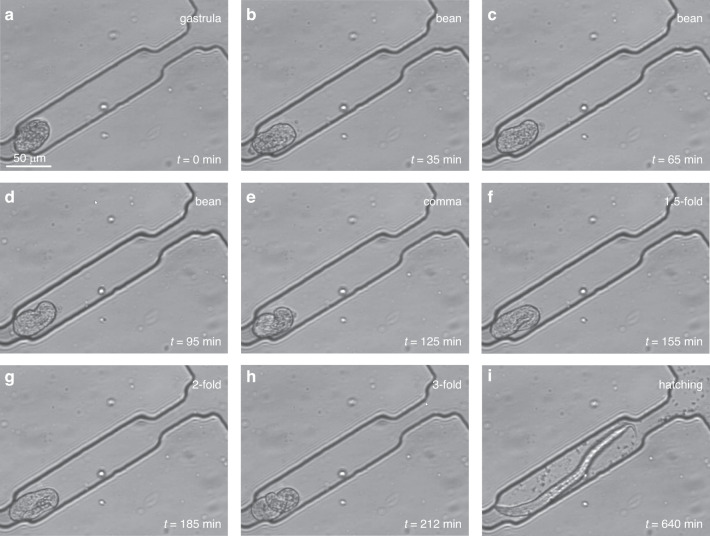
Images showing the embryonic development from the gastrula stage to the hatching stage in the M9 buffer at zero flow rate. **a** Gastrula stage. **b**–**d** Bean stage. **e** Comma stage. **f** 1.5-fold stage. **g** 2-fold stage. **h** 3-fold stage. **i** Hatching stage

To test whether wild-type *C. elegans* embryos develop normally in the microfluidic device, the embryogenesis of captured embryos was monitored through live imaging until hatching. Figure 4 is a sequence of representative bright-field images (20×, NA = 0.45) of a wild-type embryo captured in the side cavity at zero flow rate after the mixed population of *C. elegans* was loaded into the spiral channel, and single embryos were captured (Fig. S5). Several key events of wild-type *C. elegans* embryogenesis can be seen in Fig. 4, including the gastrula stage (Fig. 4a), the bean stage (Fig. 4b–d), the comma stage (Fig. 4e), the 1.5-fold stage (Fig. 4f), the 2-fold stage (Fig. 4g), the 3-fold stage (Fig. 4h), and the hatching stage (Fig. 4i). At the 3-fold stage, embryonic movement inside the embryo became active (Video S3), and pharynx pumping was observed (Video S4). The observed embryonic development and hatching events confirmed that our microfluidic device allowed long-term live imaging of *C. elegans* embryos and provided detailed visualization of the development process. It should be noted that in the static M9 buffer at 23 °C, it took ~420 min for a wild-type embryo to hatch from the 3-fold stage (Fig. 4h, i), which is slightly longer than reported values in the flowing M9 buffer^34^. The experimental results indicate that hydrodynamic forces may affect the growth and activity of *C. elegans* in the embryonic stage.

### Flow rate effect on *C. elegans* embryo development

All organisms and cells are subjected to physical and chemical stimuli during their life cycle. Among the various forms of stimulation, mechanical stimulation has been reported to affect the proliferation and differentiation of cells^35^. Although mechanical sensation is important for organism development and survival^36^, few studies on the effect of mechanical stimuli at the organismal level have been reported. There exist several types of mechanical stimuli for organisms, such as localized touch, surface texture, and vibration, and organisms appear to have different types of sensors specialized in the detection of single or combined forms of mechanical stimuli^37^. Compared to localized touch and vibration, hydrodynamic force present in our case is a different form of mechanical stimulation, applied as the culture medium flows against and around the organism. To understand how different flow rates of culture medium impact *C. elegans* development, the growth of wild-type *C. elegans* embryos under different flow rates of M9 buffer in the range of 0–20 μL/min was analyzed by measuring the duration of embryo development from the initial gastrula stage to the final hatching stage (Fig. 4) at 23 °C.

**Fig. 5:**
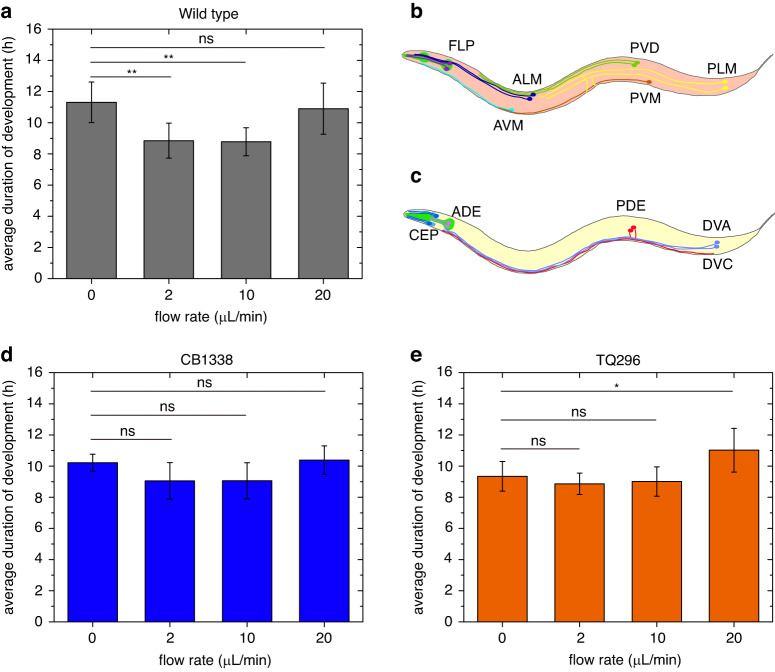
Development of wild-type C. elegans embryos and mutant strains embryos as a function of flow rate. **a** Development of wild-type embryo as a function of flow rate (*N* = 5). **b** Schematic diagram of eight mechanosensory nonciliated neurons and two ciliated neurons which are deficient in the CB1338 strain. **c** Schematic diagram of ten mechanosensory neurons which are deficient in the TQ296 strain. **d** Development of mutant strain (CB1338) embryo as a function of flow rate (*N* = 5). **e** Development of mutant strain (TQ296) embryo as a function of flow rate (*N* = 6). *p < 0.05, **p < 0.01, and ns (not significant): *p* > 0.05

As shown in Fig. 5a, the average duration of embryo development under different flow rates was measured. The average duration of development for wild-type embryos at low flow rates (2 μL/min and 10 μL/min) significantly decreased (*p* < 0.01 by Student’s *t*-test) compared to the control group of embryos cultured in static M9 buffer (0 μL/min). The experimental results indicate that the embryos grew faster at a low flow rate than in static culture. However, when the flow rate increased to 20 μL/min, the average duration of development of wild-type embryos increased, and there was no significant change (*p* = 0.67 by Student’s *t*-test) in average duration between 20 μL/min and static culture. These results show that the flow-induced hydrodynamic force had a significant effect on the embryo’s growth speed. Gentle hydrodynamic forces produced by a low flow rate (2 μL/min and 10 μL/min) induced faster growth, which is similar to previous findings following short periods of mechanical vibration (1 h and 3 h)^36^. In comparison, the growth rate of wild-type embryos in response to a long vibration period of 9 h^36^ or a high flow rate of 20 μL/min in our study induced no significant change when compared to their control groups.

It has been reported that there are >30 mechanosensory neurons in *C. elegans*^38,39^. However, different groups of neurons are involved in sensing different types of mechanical stimulation^37,39^. To narrow down which types of neurons may be involved in the embryonic sensing of hydrodynamic forces, embryos from two mutant *C. elegans* strains deficient in different groups of mechanosensory neurons were sorted and cultured in the microfluidic device, and their response to different buffer flow rates was measured through time-lapse imaging. As shown in Fig. 5b, the first mutant strain, *mec-3(e1338)* (CB1338), has deficiencies in eight mechanosensory non-ciliated neurons (two ALM neurons, two PVD neurons, two PLM neurons, one AVM neuron, and one PVM neuron) and two ciliated FLP neurons involved in mechanosensation^40^. Among these neurons, ALM, PLM, and FLP neurons differentiate at the normal embryonic stage^14^. Thus, the CB1338 strain is deficient in four mechanosensory non-ciliated neurons (two ALMs and two PLMs) and two ciliated FLP neurons at the embryo stage. According to the experimental results shown in Fig. 5d, the average duration of development of the CB1338 embryos did not reveal significant differences (*p* > 0.05 by Student’s *t*-test) under different flow rates in the range of 0–20 μL/min, indicating that the CB1338 strain is insensitive to the applied hydrodynamic force. Thus, we inferred that the group of two ALM neurons, two PLM neurons, and two FLP neurons may be involved in the embryo’s sensing of hydrodynamic forces.

As shown in Fig. 5c, the second mutant strain, *trp-4(sy695)* (TQ296), is deficient in the TRP-4/TRPN mechanotransduction channel, which is expressed in 10 mechanosensory neurons (four CEP neurons, two ADE neurons, two PDE neurons, one DVA neuron, and one DVC neuron)^41^. Among these neurons, CEP, ADE, DVA, and DVC neurons develop normally in wild-type *C. elegans* embryos. Thus, the second mutant strain, TQ296, has deficiencies in the eight mechanosensory neurons (four CEPs, two ADEs, one DVA, and one DVC) at the embryo stage. From Fig. 5e, we can see that when the embryos were cultured at low flow rates (2 μL/min and 10 μL/min), the average duration of development of the TQ296 embryos showed no significant difference (*p* > 0.05 by Student’s *t*-test) compared to the control group of embryos cultured in static fluid. However, when the flow rate was increased to 20 μL/min, the TQ296 embryos showed a significantly longer average duration of development than the control group (*p* < *0.05* by Student’s *t*-test), indicating the effect of development inhibition by the hydrodynamic force induced by the flow rate of 20 μL/min. These results show that deficiencies in eight mechanosensory neurons (four CEPs, two ADEs, one DVA, and one DVC) made the embryos insensitive to the hydrodynamic force at relatively low flow rates. However, a large hydrodynamic force induced by a flow rate of 20 μL/min suppressed the growth of the TQ296 embryos.

### Effect of osmotic stress on *C. elegans* embryo development

The ability to survive in osmotic conditions and to repair damage induced by osmotic stress is critical for the long-term maintenance of cellular life^17,42^. Due to its unique advantages, such as a completely sequenced genome and well-established genetic manipulation methods, *C. elegans* is suitable for studying how osmotic stress is handled to uncover the associated genes and regulatory pathways^43,44^. In this study, using the microfluidic device, we examined the response of *C. elegans* embryos to different osmotic levels caused by NaCl solutions at different concentrations.

**Fig. 6:**
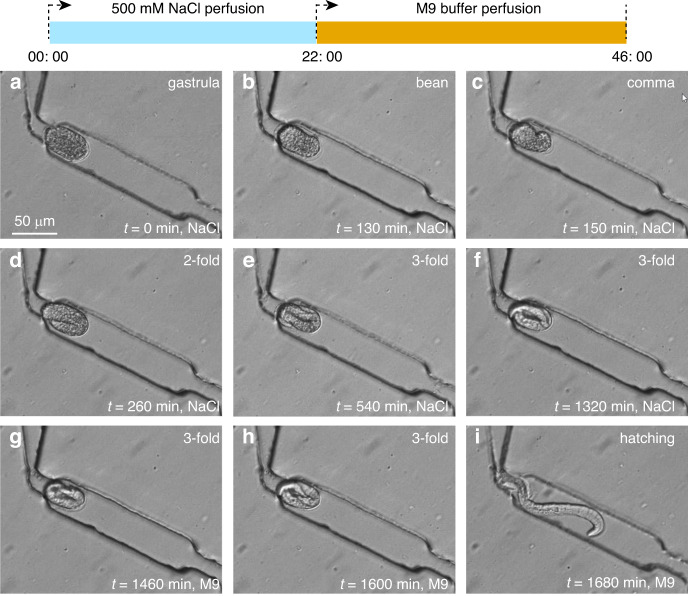
Images showing arrested N2 embryos in high-salinity solution (500 mM) can be rescued in M9 buffer. **a**–**f** Embryos cultured in the high-salinity solution for 22 h were active in mid-embryogenesis while arrested in the 3-fold stage. **g**–**i** Arrested embryos were rescued in M9 buffer

We first sorted and captured wild-type *C. elegans* embryos in the microfluidic device and then perfused the device with the NaCl solution at a flow rate of 2 µl/min for 22 h. This low flow rate allows NaCl to perfuse the cavities easily as the trapped embryos are loosened from the narrow constriction under the action of a small hydrodynamic force. After 22 h, the NaCl solution was replaced with M9 buffer, and unhatched embryos were continuously cultured in M9 buffer for another 24 h. As shown in Fig. 6a–e, when cultured in 500 mM NaCl solution, the embryos first developed from the gastrula stage to the 3-fold stage within 9 h (540 min), but embryonic development then stopped at the 3-fold stage. From Fig. 6f, one can see that after 22 h (1320 min) of culture in the 500 mM NaCl solution, the embryos were not able to hatch into the L1 stage, i.e., their development was arrested. Note that the average time period for a wild-type embryo to develop from the gastrula stage to the L1 stage was 8.85 h at a flow rate of 2 µL/min, as shown in Fig. 5. To confirm whether this arrested state was reversible, we replaced the NaCl solution with M9 buffer to further perfuse the arrested embryo. We found that 6 h after the replacement of NaCl with M9 perfusion (from Figs. 6f to 6i), the embryos were successfully hatched into the L1 stage. Furthermore, when a 3-fold embryo was arrested, it showed no movement. After the medium was replaced with M9 buffer, the embryos started to show both body movements and pharynx pumping within 260 min (Video S5).

**Fig. 7:**
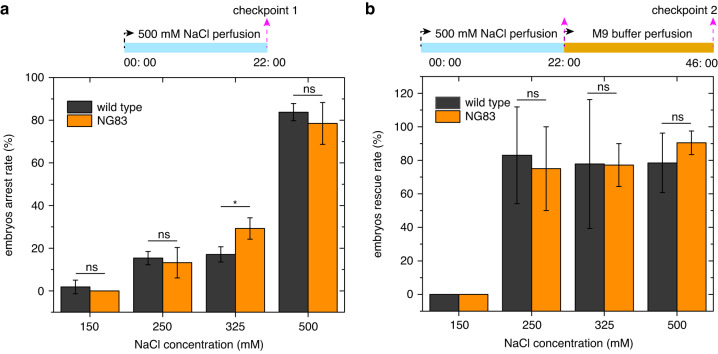
Dose response of *C. elegans* embryos in the NaCl solution and in the M9 buffer. **a** Percentage of arrested wild-type embryos and mutant embryos (*fax-1(gm83)*) as a function of concentration of NaCl solution when the embryos were cultured in the NaCl solution for 22 h (*N* = 3 devices). **b** Percentage of rescued wild-type embryos and mutant embryos (*fax-1(gm83)*) in M9 buffer for another 24 h after being cultured in different concentration of NaCl solution for 22 h (*N* = 3 devices). *p < 0.05, ns (not significant): *p* > 0.05

We also adjusted the NaCl concentration to study its effect on embryo development. As shown by the black bars in Fig. 7a, we quantified the percentage of arrested wild-type embryos on the same device after 22-h perfusion of NaCl at concentrations of 150–500 mM. At 150 mM, no more than one embryo per batch (in the same device) was arrested, and the percentage of arrested embryos was only 1.85 ± 3.21%. When the NaCl concentration was increased to 250 mM and 325 mM, the average percentage of arrested embryos rose to 15.39 ± 3.14% and 17.08 ± 3.61%, respectively. At 500 mM, the percentage of arrested embryos reached 83.78 ± 4.02%, which is much higher than the percentage observed in embryos cultured at lower NaCl concentrations. To determine how many arrested embryos could be rescued, the NaCl solutions at different concentrations were then replaced with M9 buffer, and the arrested embryos were cultured for another 24 h. As shown in Fig. 7b (black columns), after another 24 h of culture in M9 buffer, most arrested embryos were rescued, while only a small portion of embryos remained arrested; the latter may have experienced irreversible damage from osmotic stress. These results demonstrate that the embryos arrested by osmotic stress can be rescued by the M9 buffer.

The osmotic stress response of *C. elegans* was reported to be related to insulin signaling pathways^18^. In particular, the osmotic stress pathway involves an apparent neuroendocrine signal produced from sensory neurons that favors the developmental arrest of *C. elegans*^18^. *C. elegans* can prevent itself from entering the state of arrested development through insulin signaling regulation^18^. To investigate whether a mutant strain with reduced insulin signaling could increase the severity of the embryonic arrest phenotype, the mutant strain *fax-1(gm83)* (NG83) was selected for the same osmotic stress experiment. *fax-1* is a transcription factor gene that encodes a nuclear hormone receptor that potentiates insulin signaling. Loss of the *fax-1* gene decreases insulin signaling.

The NG83 mutant embryos were first sorted and captured in the microfluidic device and then perfused with different concentrations of NaCl at a flow rate of 2 µl/min for 22 h. After that, the arrested embryos were continuously cultured in M9 buffer for another 24 h. As shown in Fig. 7a (orange bars), the percentage of arrested mutant embryos increased with the concentration of NaCl solution. At 150 mM, no mutant embryos in any batch (in the same device) were arrested. When the concentration of NaCl was increased to 250 mM and 325 mM, the average percentage of arrested mutant embryos rose to 13.23 ± 7.14% and 29.26 ± 5.01%, respectively. At a concentration of 500 mM, the percentage reached 78.50% ± 9.84%, which is much higher than that of the mutant embryos cultured at lower NaCl concentrations. Notably, at 150 mM, 250 mM, and 500 mM, the arrest rate of NG83 embryos showed no significant difference from that of the wild-type embryos (*p* > *0.05* by Student’s *t*-test). However, at a concentration of 325 mM, there was a significant difference in the arrest rate between the N83 mutant embryos and the wild-type embryos (*p* = 0.027 by Student’s *t*-test). These results indicate that the NG83 embryos (with reduced insulin signaling) had increased sensitivity to embryonic arrest at the modest NaCl concentration of 325 mM. This phenomenon was also observed in a previous study in which mutant embryos were cultured on NGM plates with different concentrations of NaCl^18^.

When the arrested mutant embryos were cultured in M9 buffer for another 24 h, most of the arrested embryos were rescued (orange bars in Fig. 7b). The average rescue rates of the mutant embryos that were arrested at 250 mM, 325 mM, and 500 mM were 75 ± 25%, 77.18 ± 12.77%, and 90.45 ± 7.05%, respectively. It should be noted that no significant change was observed between the rescued percentages of mutant embryos and wild-type embryos when they were pre-arrested in the same concentration of NaCl (*p* > *0.05* by Student’s *t*-test; Fig. 7b). Overall, the results in Fig. 7 show that reduced insulin signaling affected the sensitivity of the mutant strain to osmotic arrest, especially under mild osmotic stress, but it did not significantly change the rescued percentage of arrested embryos when cultured in M9 buffer. Taken together, the evidence illustrates how our microfluidic device can be employed to study the response of trapped *C. elegans* embryos to chemical stimuli and investigate developmental mechanisms.

## Conclusion

In summary, we have developed an easy-to-use microfluidic device for effective sorting, trapping, and long-term imaging of *C. elegans* embryos. The principle of embryo sorting from a mixed population of *C. elegans* relies on the Dean vortices generated in the cross-section of a spiral channel, and embryo trapping inside side cavities at single embryo resolution depends on passive hydrodynamics. When compared to a straight channel, the spiral channel introduces Dean vortices that allow the effective separation of embryos from L4/adult worms and enable single embryos to be isolated quickly from a mixture of *C. elegans* due to the mixing effect of secondary flows. We successfully trapped embryos at single embryo resolution with a success rate up to 85% at a flow rate of 1 mL/min. Using this device, it was found that the embryos were sensitive to the flow rate, and their average duration of embryonic development from the gastrula stage to the hatching stage differed under different flow rates. Two mutant strains were selected for mechanotransduction studies, and two different broad groups of mechanosensory neurons were found to be involved in sensitivity to hydrodynamic forces at the embryonic stage. Through the well-controlled chemical microenvironment surrounding the trapped embryos, the response of the embryos to osmotic stress was observed. Interestingly, it was found that the arrested embryos in high-salinity solution could be rescued when these arrested embryos were cultured in M9 buffer. Additionally, the mutant strain with reduced insulin signaling was found to be more sensitive to the high-salinity solution. These experiments showed that the microfluidic device can be used to sort and trap single *C. elegans* embryos and control the microenvironment surrounding the trapped embryos for a multitude of biological studies.

## Materials and methods

### Preparation of *C. elegans*

*C. elegans* worms were cultured on nematode growth medium (NGM) agar^45^, which was prepared by dissolving 2.5 g of peptone, 17 g of agar, and 3 g of NaCl in 1 L distilled water, followed by a 1 h autoclave. After that, the autoclaved mixture was placed in a 55 °C water bath for 1 h to cool down. Then, 1 mL of 1 M CaCl_2_, 1 mL of 1 M MgSO_4_, 25 mL of 1 M potassium phosphate buffer (pH 6.0), and 1 mL of 5 mg mL^-1^ cholesterol were mixed with the autoclaved solution to prepare the final NGM agar plate. The final solution was then poured on a petri dish and seeded with *E. coli* OP50 bacteria as food. The M9 buffer for *C. elegans* culture in the microfluidic device was made by adding 6 g of Na_2_HPO_4_, 3 g of KH_2_PO_4_, and 5 g of NaCl into 1 L distilled water and autoclaving the mixture. After cooling, 1 mL of autoclaved 1 M MgSO_4_ was added to the mixture to obtain the final M9 buffer.

Both wild-type and transgenic worms were tested using the proposed microfluidic device. The worm strains used were N2 Bristol (wild-type), strain CB1338 (*mec-3(e1338)*), strain TQ296 (*trp-4(sy695)*), and strain NG83 (*fax-1(gm83)*). The transgenic strains were obtained from the Caenorhabditis Genetics Center (CGC). The mixed population of worms used in single-embryo sorting and trapping consisted of embryos, L1 larvae, L4 larvae, and adult worms. This mixed population was obtained by the following protocol. First, 5-8 adult worms were cultured on a fresh agar plate (with OP50) for 1 day. Later, these 5–8 adult worms were removed, and the remaining embryos or larval worms were continuously maintained for another two and one-half days at 21 °C. Finally, the mixed population consisting of a large number of embryos, L1 larvae, L4 larvae, and adult worms was collected by washing off the agar plates using a pipette and M9 buffer (Fig. S4 and Table S1). The mixed population suspended in the M9 buffer was collected in a 10 mL syringe and then loaded into the microfluidic device.

### Experimental setup

To achieve clear long-term live imaging of embryos captured in the side cavities, the microfluidic device was mounted on the motorized X-Y stage (ProScan III, Prior) of an inverted microscope (IX83, Olympus), which positioned embryos under the field of view of the microscope. A computer-controlled syringe pump was employed to regulate the flow rate at the inlet of the microfluidic device. Clear imaging was obtained through a complementary metal-oxide semiconductor (CMOS) camera (acA2000-340 km, Basler; 2040 × 1080 pixels) mounted on the microscope, and a host computer was responsible for running the custom-made control software for syringe pump regulation.

### Fabrication of the microfluidic device

The microfluidic device consists of two main components: the top PDMS layer, including the spiral channel and 20 side cavities uniformly distributed along the spiral channel, and a glass slide coated with a thin PDMS layer. The spiral channel is 1.6 mm wide and 53 μm high. The microfluidic device was fabricated through multilayer soft lithography. First, SU-8 2050 photoresist (Microchem) with a thickness of 53 μm was spin-coated onto a silicon wafer and then patterned into the SU-8 mold of the top PDMS layer by standard photolithography. This mold was further treated with tridecafluoro-1,1,2,2-tetrahydrooctyl-1-trichlorosilane via chemical vapor deposition for 1 h to render its surface hydrophobic. After that, a precursor mixture of PDMS base and cross-linker (Sylgard 184, Dow Corning) at a w/w ratio of 10:1 was poured onto the SU-8 mold and cured at 80 °C for 120 min. The PDMS layer was peeled from the SU-8 molds and punched with open inlet and outlet holes. Then, a thin PDMS layer with a thickness of 20 μm was spin-coated on a glass slide (75 × 50 × 1.6 mm^3^) using the same PDMS precursor (w/w mixing ratio: 10:1). The glass slide was cured at 80 °C for 120 min. Finally, the PDMS layer and glass slide were bonded together to form the microfluidic device. As schematically shown in Fig. S1, the width of the narrowest part of the side cavity is 15 μm, and the height of the narrowest part is 53 μm. Although the aspect ratio of the narrowest part of the side cavity is high (~3.5), the fabrication of the SU-8 mold for the whole microfluidic device was always successful. This might be because the narrowest part is short (25 μm) and is fully connected to thick structures with low aspect ratios.

### Supplementary information

Supplementary material

Video S1-embryo sorting from mixed population of C.elegans

Video S2-Single embryo trapping inside side cavity

Video S3-long term live imaging of C. elegans embryos cultured in M9 buffer

Video S4-pharyngeal pumping observed at 3-fold stage of C.elegasn embryos

Video S5-Osmotic stress response of C.elegasn embryos
