# Correction: A spiral microfluidic device for rapid sorting, trapping, and long-term live imaging of *Caenorhabditis elegans* embryos

**DOI:** 10.1038/s41378-023-00626-9

**Published:** 2023-11-24

**Authors:** Peng Pan, Zhen Qin, William Sun, Yuxiao Zhou, Shaojia Wang, Pengfei Song, Yong Wang, Changhai Ru, Xin Wang, John Calarco, Xinyu Liu

**Affiliations:** 1 Department of Mechanical and Industrial Engineering, University of Toronto, 5 King’s College Road, Toronto, ON M5S 3G8 Canada; 2 Upper Canada College, 200 Lonsdale Road, Toronto, ON M4V 1W6 Canada; 3 School of Advanced Technology, Xi’an Jiaotong-Liverpool University, 111 Ren’ai Road, Suzhou, 215000 China; 4 School of Electronic and Information Engineering, Suzhou University of Science and Technology, Suzhou, 215009 China; 5 Department of Mechanical and Aerospace Engineering, Jilin University, Changchun, 130012 China; 6 Department of Cell & Systems Biology, University of Toronto, 25 Harbord St, Toronto, ON M5S 3G5 Canada; 7 Institute of Biomedical Engineering, University of Toronto, 164 College Street, Toronto, ON M5S 3G9 Canada

**Keywords:** Engineering, Materials science

Correction to: *Microsystems & Nanoengineering* (2023) **9**:17

10.1038/s41378-023-00485-4 published online 21 February 2023

After the publication of this article^1^, it was brought to our attention that a fund number in the acknowledgements needs to be updated:

**Correction 1:**

This work was supported by the Natural Sciences and Engineering Research Council of Canada (grant numbers: RGPIN-2017-06374, RGPAS-2017-507980, and RGPIN-2022-05039), the Canadian Institutes of Health Research (grant number: PJT-180365), the Canada Foundation for Innovation (grant number: JELF-38428). The financial support from the National Natural Science Foundation of China (62273247) and the Natural Science Foundation of the Jiangsu Higher Education Institutions of China (20KJA460008) to C. Ru is acknowledged. P. Song also acknowledges the support from the Natural Science Foundation of the Jiangsu Higher Education Institutions of China (20KJB460024) and the Young Scholar Program of Jiangsu Science and Technology (BK20200251).
