# Supplementary material for: A spiral microfluidic device for rapid sorting, trapping, and long-term live imaging of Caenorhabditis elegans embryos
Source: Microsyst Nanoeng. 2023 Feb 21;9:17. doi: 10.1038/s41378-023-00485-4 (PMC9943735; doi:10.1038/s41378-023-00485-4)
Supplement: Supplementary file 1 — Supplementary material [file 41378_2023_485_MOESM1_ESM.docx]

**Supporting Information**

**A spiral microfluidic device for rapid sorting, trapping, and long-term live imaging of *Caenorhabditis* *elegans* embryos**

Peng Pan^1^, Zhen Qin^1^, William Sun^2^, Yuxiao Zhou^1^, Shaojia Wang^1^, Pengfei Song^3^, Junhui Zhu^4^, Changhai Ru^4^, Xin Wang^5^, John Calarco^6^, Xinyu Liu^1,7*^

^1^ Department of Mechanical and Industrial Engineering, University of Toronto, 5 King’s College Road, Toronto, Ontario, M5S 3G8, Canada

^2^ Upper Canada College, 200 Lonsdale Road, Toronto, Ontario, M4V 1W6, Canada

^3^ School of Advanced Technology, Xi'an Jiaotong-Liverpool University, 111 Ren’ai Road, Suzhou, 215000, China

^4^ School of Electronic and Information Engineering, Suzhou University of Science and Technology, Suzhou, 215009, China

^5^ Department of Mechanical and Aerospace Engineering, Jilin University, Changchun, 130012, China

^6^ Department of Cell & Systems Biology, University of Toronto, 25 Harbord St, Toronto, Onatrio, M5S 3G5, Canada

^7^ Institute of Biomedical Engineering, University of Toronto, 164 College Street, Toronto, Onatrio, M5S 3G9, Canada

*Email: [xyliu@mie.utoronto.ca](mailto:xyliu@mie.utoronto.ca)

1. **Microfluidic device design**


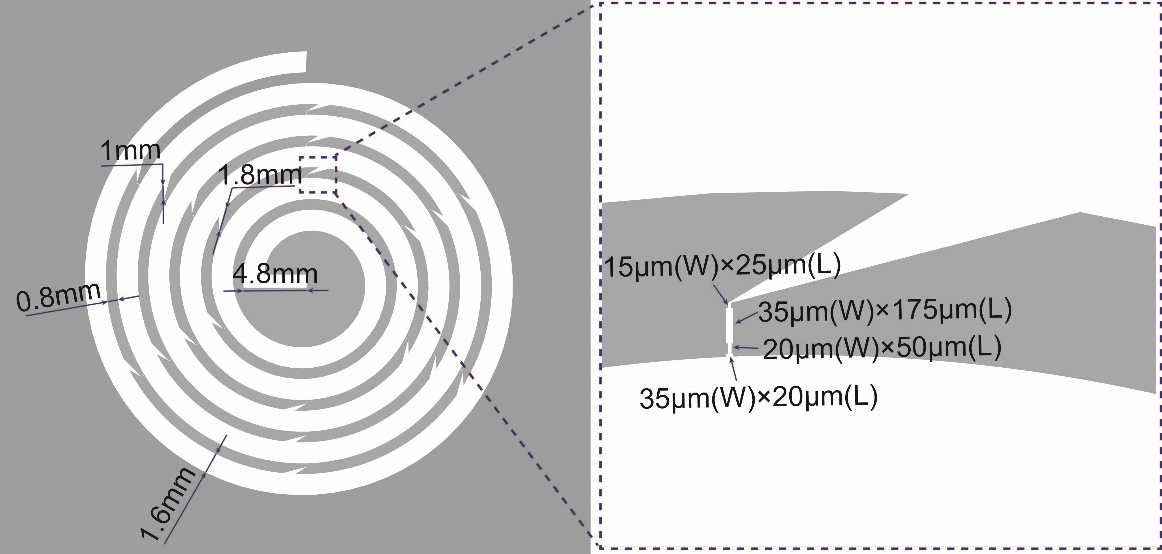


***Figure S1.*** *Detailed information of microfluidic channel.*

This microfluidic device is composed of a spiral channel for embryos sorting and 20 uniformly distributed side cavities for single embryos trapping and long-term live imaging. The height of fabricated microfluidic channel is 53 μm. The other dimension of microfluidic channel and side cavity is shown in **Figure S1**.

1. **Volume flow rate analysis**


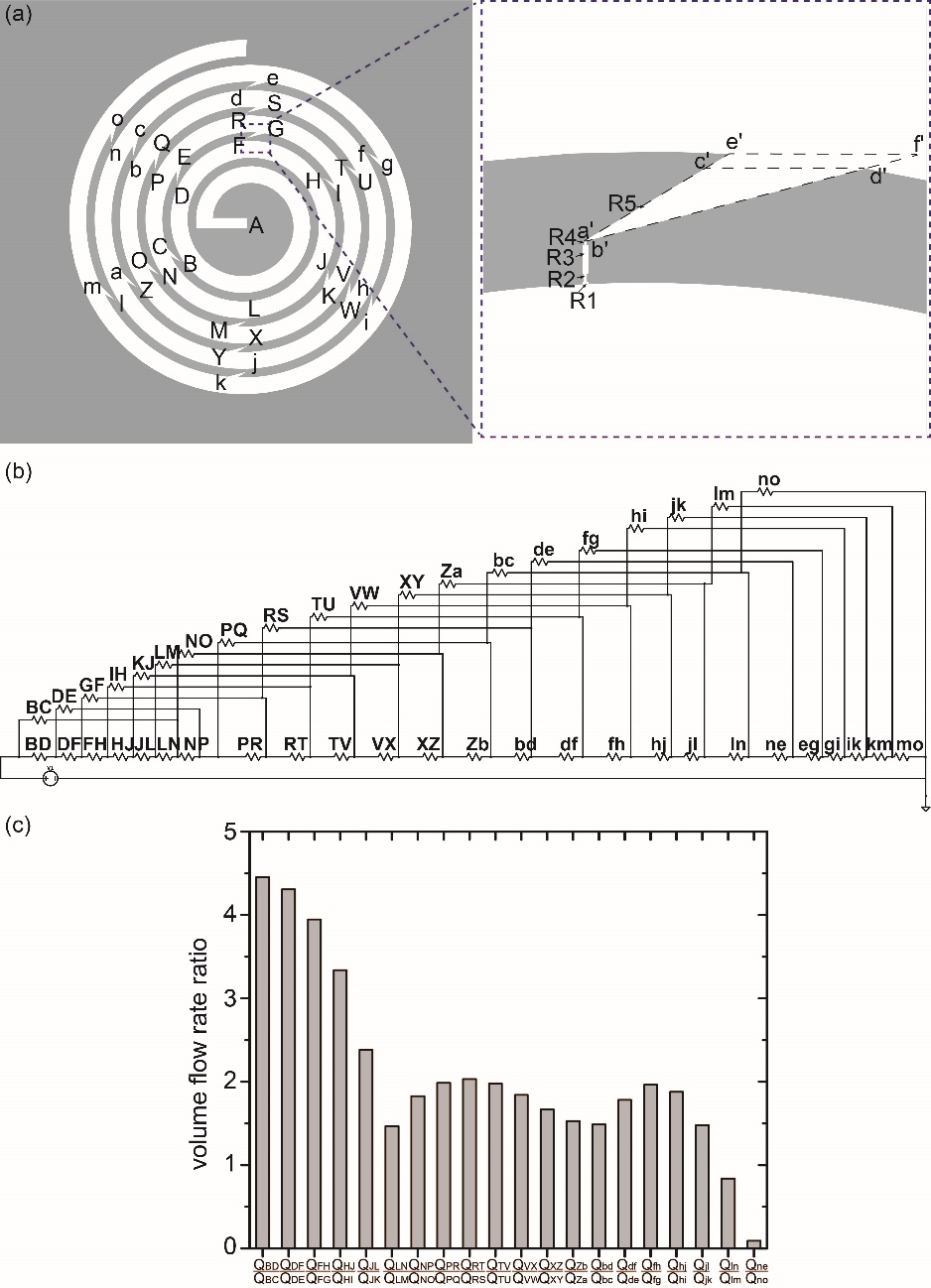


***Figure S2.*** *Analysis of volume flow rate for the microfluidic channel.*

The microfluidic chip is proposed to sort embryos from the mixed population (consists of embryos, L1, L4, and adult worms) and capture the focused embryos at single embryo resolution. Under the action of dean drag and lift forces, embryos are focused close to the outer wall of channel. L4/adult worms which have large hydraulic diameters are focused close to the inner wall. As the spiral channel has a relatively large width of 1.6 mm, to ensure that focused embryos can be successfully captured by the side cavity while L4/adult worms focused close to the inner wall can easily move downstream, the volume flow rate along the side cavity should be smaller than or close to that of corresponding main channel part. For example, volume flow rate along the side cavity “NO” (*Q_NO_*) should be smaller than or close to that along the main channel part “NP” (*Q_NP_*).

To optimize the microfluidic device design, volume flow rates along the side cavities and their corresponding main channels parts were analyzed. Whole spiral channel was divided into different parts, shown in the **Figure S2a**. For each part, it has different hydraulic resistance. The whole microfluidic channel is equivalent to an electric circuit for the analysis of volume flow rate. To simplify the circuit, it is assumed that the fluidic pressure at the closet points from two adjacent side cavities (such as “E” and “P”, “G” and “R”) are same. The equivalent circuit is displayed in the **Figure S2b**. **Figure S2c** shows the volume flow rate ratio of the main channel part to the corresponding upstream side cavity with finalized microfluidic channel design. As shown in the **Figure S2c**, most volume flow rate ratios are larger than or close to 1, and this ensures L4/adult worms could be focused close to the inner wall of spiral channel and easily bypass the traps. It should be noted that only the volume flow rate along the last trap (“no”) and its corresponding main channels parts (*Q_ne_/Q_no_)* is relatively small which is ~0.1. However, due to the long-term focusing of L4/adult worms at the inner side of spiral channel and large width of spiral channel, the probability that last trap (“no”) capture L4/adult worms is very small. This is consistent with experimental observation. With the optimized microfluidic channel, we have obtained the singe embryo capturing with high success rate.

The calculation of hydraulic resistance of each side cavity is described as follows. Each side cavity is divided into five parts which are connected in series, as shown in **Figure S2a**. Thus, hydraulic resistance of side cavity (*R_sd_*) is the sum of each separated part (*R_1_, R_2_, R_3_, R_4_,* and *R_5_*). The expression of hydraulic resistance formula is^1^:

$$R=C(\alpha)\frac{\mu L}{2D^{2}A}$$

Where $C(\alpha)\text{=96(1-1.3553}\alpha+1.9467\alpha^{2}\text{-1.7012}\alpha^{3}\text{+0.9564}\alpha^{4}\text{-0.2537}\alpha^{5}\text{)}$and *α is the* aspect ratio which is defined as either height/width or width/height such that 0≤ *α* ≤1^1^. *L* is the length of microfluidic channel, *D* is the hydraulic diameter, *A* is the cross-sectional area of microfluidic channel, and $\mu$is the fluid viscosity. After calculation, the hydraulic resistance *R_1_*, *R_2_*, *R_3_*, and *R_4_*, were $1.81\mu\times10^{5}$, $18.6\mu\times10^{5}$, $15.9\mu\times10^{5}$, and $20.4\mu\times10^{5}$. For the hydraulic resistance *R_5_*, it is larger than *R_a’b’c’d’_*, but smaller than the *R_a’b’e’f’_* **(Figure S2a***)*. *R_a’b’c’d’_* is calculated to be $4.34\mu\times10^{5}$and *R_a’b’e’f’_* is calculated to be $4.40\mu\times10^{5}$. As the difference between *R_a’b’c’d’_* and *R_a’b’e’f’_* can be neglected when compared to *R_a’b’c’d’_,* we assume that the hydraulic resistance *R_5_* is equal to *R _a’b’c’d’._* Finally, the hydraulic resistance of each side cavity is obtained which is $6.136\mu\times10^{6}$. The hydraulic resistance of corresponding part of main channel can be easily obtained according to the equation mentioned above. For example, the hydraulic resistance of spiral channel parts *R_BD_, R_DF_* and *R_FH’_are calculated to be* $3.56\mu\times10^{5}$, $3.76\mu\times10^{5}$, $3.96\mu\times10^{5}$, respectively.

In theory, more than 300 side cavities can be designed along the microfluidic channel to capture the embryos focused close to the outer wall. If more side cavities are required, the resistance of side cavities should be increased or the resistance of spiral channel should be decreased by adjusting their size correspondingly.

1. **Simulation analysis**


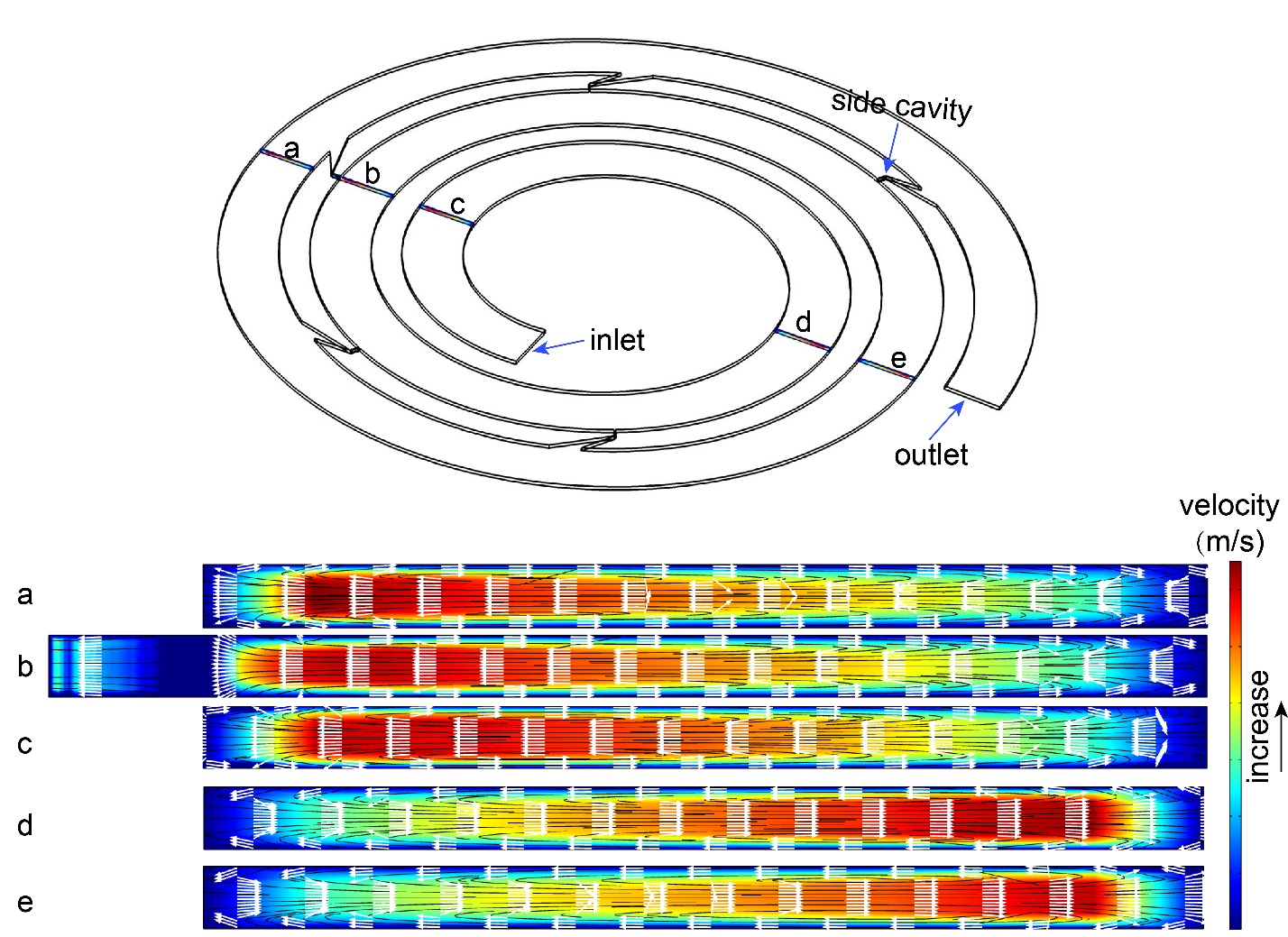


***Figure S3.*** *Stream pattern simulation in the cross-section of spiral channel at different positions.*

To demonstrate that the Dean vortex can generate in cross-section when side cavities are designed along the spiral channel, numerical analysis was performed, as shown in **Figure S3**. From **Figure S3**, we can see Dean vortex can still successfully generate. Even in the cross-section close to the side cavity (**Figure S3b**), Dean vortex is observed.

1. **Mixed population of *C. elegans***


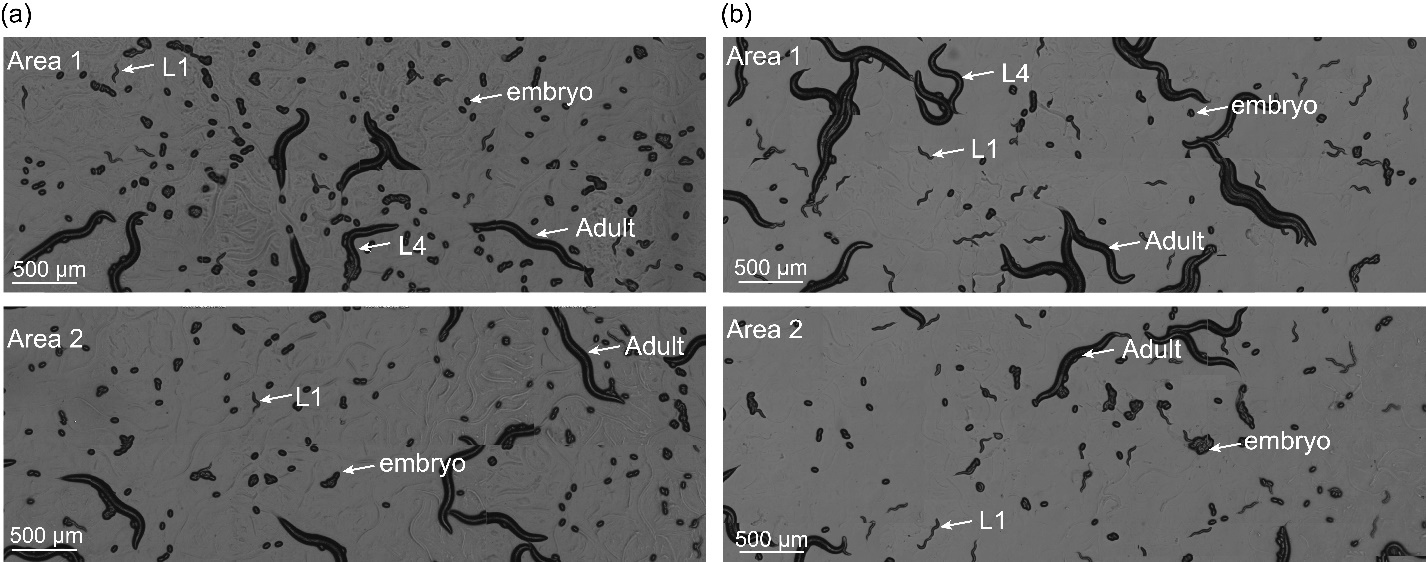


***Figure S4.*** *Mixed population of worms consisting of only embryos, L1, L4, and adults are observed from different areas of two different NGM plates. (a) images of two different areas (Area 1 and Area 2) from one NGM plate. (b) images of two different areas (Area 1 and Area 2) from another NGM plate*

The mixed population of worms used in the single embryos sorting and trapping consists of embryos, L1, L4, and adult worms. This mixed population were obtained by the following protocol. First, 5-8 adult worms were cultured on a fresh agar plate (with OP50) for one day. Later, these 5-8 adult worms were removed, and left embryos or larval worms were continuously maintained for another two and half days at 21 °C. Finally, the mixed population consisting of a large number of embryos, L1, L4 and adult worms was obtained **(Figure S4)**. Mixed population of worms were imaged from different areas of two plates as shown in **Figure S4(a) and (b).** Number of worms at different developmental stages in different NGM plates were counted, as shown in the **Table S1**.

**Table S1.** Number of worms at different developmental stages in each NGM plate

| **NGM Plate #** | **Total number of Adults** | **Total number of L4** | **Total number of L3** | **Total number of L2** | **Total number of L1** | **Number of embryos** |
| --- | --- | --- | --- | --- | --- | --- |
| **1** | **217** | **10** | **0** | **0** | **1172** | **4100** |
| **2** | **267** | **5** | **0** | **0** | **205** | **5300** |
| **3** | **165** | **6** | **0** | **0** | **450** | **4450** |
| **4** | **198** | **1** | **0** | **0** | **458** | **5980** |

1. **Image of whole microfluidic device**

**
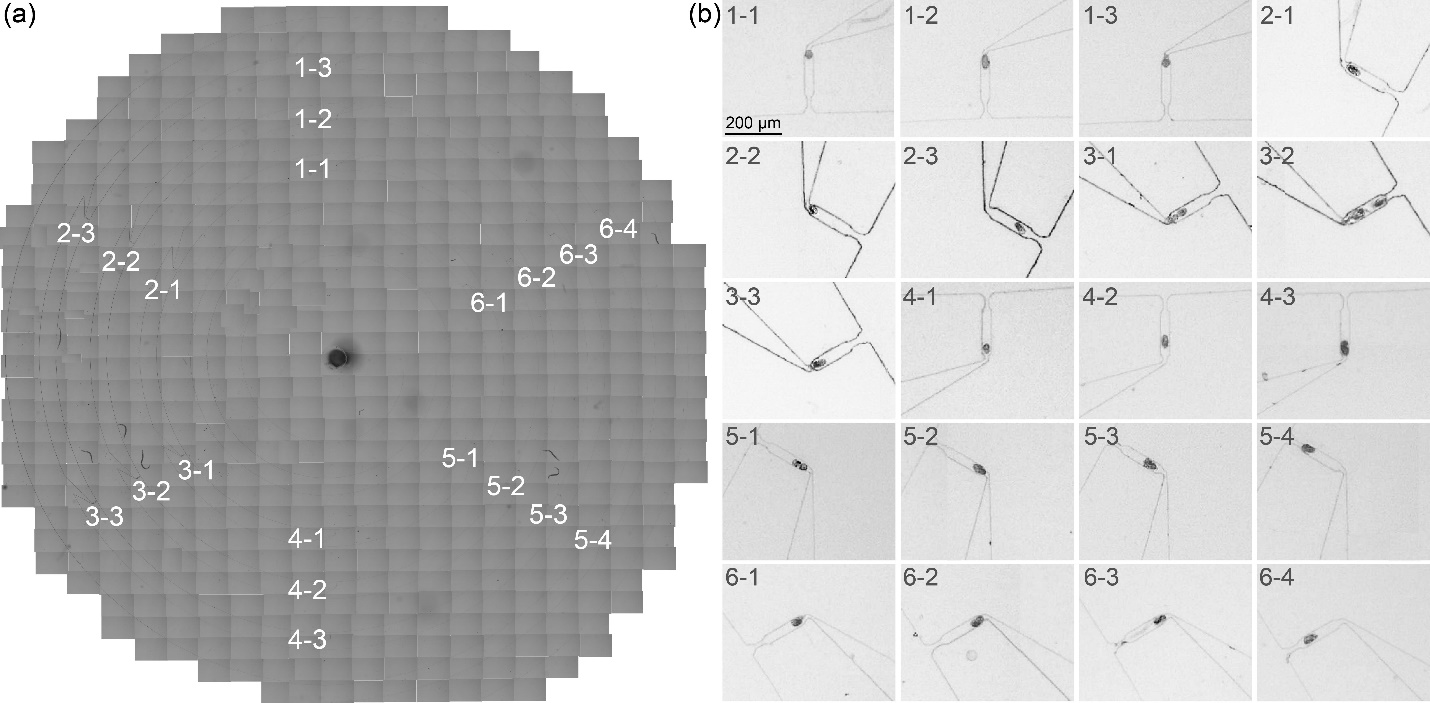
**

***Figure S5.*** *Images showing embryo trapping inside the side cavities of microfluidic device. (a) Stitched images showing the whole microfluidic device. (b) Enlarged view of each side cavity.*

To demonstrate that the proposed microfluidic device can be employed to capture embryos at single-embryo resolution, the mixed population of worms mainly consisting of embryo, L1, L4, and adult worms were loaded into the microfluidic channel at the flow rate of 1 mL/min. After all side cavities captured embryos, the microfluidic device was mounted on the inverted microscope and scanned under 4$\times$objective. Finally, all scanned images were stitched together to show the whole microfluidic device, as shown in **Figure S5a**. **Figure S5b** shows the enlarged view of each side cavity. From **Figure S5b**, we can see 17 of 20 side cavities captured single embryo which provides accurate long-term live imaging of the embryos and only three cavities (marked as 3-1, 3-2, and 5-1) captured two embryos. This device shows huge potential in high-content screening of *C. elegans* embryos.

1. **Reference**
2. Tan, W. H. & Takeuchi, S. A trap-and-release integrated microfluidic system for dynamic microarray applications. *Proc. Natl. Acad. Sci. U. S. A.* **104**, 1146–1151 (2007).
